# Quantitative Antioxidant Profiling Throughout Beer Brewing Followed by Discovery and Isolation of Precursors from Barley (*Hordeum vulgare* L.)

**DOI:** 10.1021/acs.jafc.4c00998

**Published:** 2024-06-04

**Authors:** Stefan Spreng, Julia Wannenmacher, Martina Gastl, Corinna Dawid, Thomas Hofmann

**Affiliations:** † Chair of Food Chemistry and Molecular and Sensory Science, Technical University of Munich, Lise-Meitner-Str. 34, D-85354 Freising, Germany; ‡ Chair of Brewing and Beverage Technology, Technical University of Munich, Weihenstephaner Steig 20, D-85354 Freising, Germany; § Bavarian Center for Biomolecular Mass Spectrometry, Gregor-Mendel-Straße 4, D-85354 Freising, Germany

**Keywords:** antioxidants, beer, brewing process, hordatines, phenols, quantification, tachioside, barley, Hordeum vulgare

## Abstract

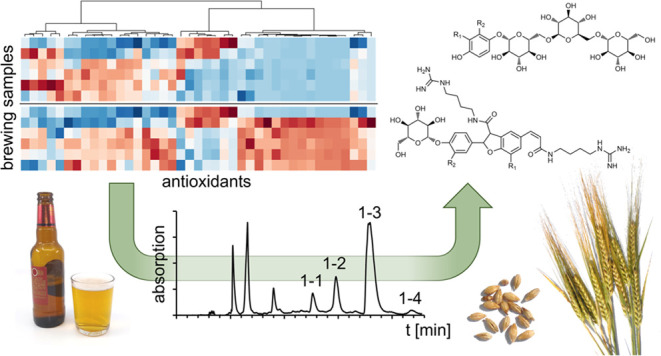

The application of high-performance liquid chromatography-mass spectrometry (HPLC-MS/MS) revealed the origin and evolution of antioxidants during the brewing process of hopped and unhopped reference beer. As tachioside (3-methoxy-4-hydroxyphenyl-β-d-glucopyranoside), arbutin (4-hydroxyphenyl-β-d-glucopyranoside), and hordatines clearly increased during the fermentation step, the raw material barley was investigated as a source of the corresponding precursors. Therefore, 4-hydroxyphenyl-β-d-glucopyranosyl-(1 → 6)-β-d-glucopyranosyl-(1 → 6)-β-d-glucopyranoside, 4-hydroxy-3-methoxyphenyl-β-d-glucopyranosyl-(1 → 6)-β-d-glucopyranoside, 4-hydroxy-3-methoxyphenyl-β-d-glucopyranosyl-(1 → 6)-β-d-glucopyranosyl-(1 → 6)-β-d-glucopyranoside, and 4-hydroxy-2-methoxyphenyl-β-d-glucopyranosyl-(1 → 6)-β-d-glucopyranosyl-(1 → 6)-β-d-glucopyranoside were isolated from barley for the first time, and identified using liquid chromatography–mass spectrometry (LC–MS) and one-dimensional/two-dimensional-nuclear magnetic resonance (1D/2D-NMR) experiments. Moreover, hordatine glucosides A, B, and C were isolated and identified from barley, and hordatine C glucoside was characterized for the first time. A fermentation model followed by HPLC-MS/MS analysis substantiated the release of tachioside from 4-hydroxy-3-methoxyphenyl-β-d-glucopyranosyl-(1 → 6)-β-d-glucopyranoside by *Saccharomyces cerevisiae*. Quantitation experiments monitoring the content in wheat, barley, and different barley malt types demonstrated a wide range of concentrations, providing a basis for further comprehensive investigations to optimize the antioxidant yield in beer to contribute to improved flavor stability.

## Introduction

Among consumers, beer is admired because of its unique aroma and taste profile. The flavor stability of beer, however, is still one of the main factors limiting its shelf life.^1−3^ Many investigations have been performed on the sensory profile of fresh and aged beer to unveil the molecular principles.^4−8^ As oxygen seems to be of crucial importance for the reactions occurring during storage, antioxidants are said to be appropriate to slow down the aging process.^1^ Thereby, especially naturally occurring antioxidants came into focus, as local regulations limit the use of additives, and consumers prefer products in their natural state.^9^

Various compound classes, such as sulfites, Maillard-reaction products, and polyphenols have already been demonstrated to be antioxidants in beer,^1^ and very recently, an activity-guided fractionation approach revealed a number of additional compounds that were not previously considered antioxidants in beer.^10^ Though hordatines, known from barley,^11^ have been suggested to have an impact on the astringent taste of beer,^12^ recently they have been identified unequivocally by isolating them from beer, and strong antioxidant properties have been highlighted.^10^ Due to the health benefits of antioxidants, different side products of barley, such as brewer’s spent grain^13^ and barley leaves,^14,15^ have been found to contain a large variety of phenolic compounds. Some of these compounds, including saponarin, syringaresinol, and feruoylquinic acid, have also been reported in beer. Although the contribution from hops and malt can be well distinguished for many phenolic compounds,^16^ even the origin of some antioxidants, such as tachioside, remains unknown. Concerning the evolution during production, mainly the influence of wort boiling has been studied, both for selected phenolics^17,18^ and oxidized thiols.^19^ A partial release during fermentation was reported, and, for ferulic acid, feruloyl esterase activity^20^ and selected phenolic constituents were tracked in starting and spent beer ingredients.^21^ However, a more holistic view of the brewing process is needed to identify crucial steps for antioxidant intake, similar to what has been performed in the case of hop-derived bitter constituents in beer.^22^ Hence, the aim of this study was to track the evolution of the antioxidants in beer during the brewing process by liquid chromatography with tandem mass spectrometry (LC–MS/MS)_MRM_. An additional set of unhopped reference samples was arranged to obtain hints about the antioxidants’ origin and potential precursors to further aim for targeted isolation of the suggested precursors in the raw material using preparative high-performance liquid chromatography (HPLC). The purified compounds were intended to both achieve unambiguous elucidation of their chemical structure by means of LC-MS and nuclear magnetic resonance (NMR) experiments and to allow the analysis of their content in the raw material by LC–MS/MS_MRM_. These extensive insights will enable the derivation of sound conclusions on how to modulate the antioxidant content and impact the flavor stability of beer.

## Materials and Methods

### Chemicals

The following compounds were obtained commercially: 2,2′-azo-bis(2-methylpropinamidine) (AAPH), fluorescein sodium salt, (±)-6-hydroxy-2,5,7,8-tetramethylchromane-2-carboxylic acid (Trolox), 2,2′-azinobis(3-ethylbenzothiazoline-6-sulfonic acid) diammonium salt (ABTS), acetic acid, (+)-catechin hydrate, disodium hydrogen phosphate, (−)-epicatechin, ethylenediaminetetraacetic acid, hemoglobine, hydrogen peroxide, iron(II)sulfate heptahydrate, peroxidase from horseradish, linoleic acid, *p*-hydroxyphenyllactic acid, pinoresinol, sodium tetraborate, syringic acid, trifluoroacetic acid (99%), 3,4,5-trimethoxybenzoic acid, triton X-100, l-tryptophan, Tween 20, l-tyrosine, tyrosol, yeast-nitrogen-base without amino acids (Sigma-Aldrich, Steinheim, Germany); caffeic acid, formic acid (98–100%), hydrochloric acid (32%), *p*-hydroxybenzoic acid, potassium dihydrogen phosphate, potassium hydroxide, 2-propanol, sodium hydroxide (Merck, Darmstadt, Germany); ammonium acetate (5 M in water), ferulic acid, fluorescein, *p*-coumaric acid, sinapic acid, vanillic acid (Fluka, Neu-Ulm, Germany); D_2_O, methanol-*d*_4_ (Euriso-Top, Saarbrücken, Germany); sodium hydroxide (Riedel-de-Haen, Seelze, Germany); *cyclo* (Pro-Tyr) (Bachem, Weil am Rhein, Germany); benzoylleucomethylene blue (TCI Europe, Zwijndrecht, Belgium); feruloyl quinic acid (mixture from green coffee), isoxanthohumol, xanthohumol (Chair of Food Chemistry and Molecular Sensory Science, Freising, Germany); apigenin, dihydrorobinetin, isorhamntein-3-*O*-glucoside (Extrasynthese, Genay, France); and l-tryptophan (indol-*d*_5_), l-tyrosine (cycle-*d*_4_) (Cambridge Isotope Laboratories, Andover). Water for HPLC separation was purified using a Milli-Q water Advantage A 10 water system (Millipore, Molsheim, France). The solvents used were HPLC-grade (J.T. Baker, Deventer, Netherlands) and MS-grade (Honeywell, Morristown, NJ), respectively. For membrane filtration, a 0.45 μm membrane filter (Sartorius, Göttingen, Germany) was used. Malting barley (variety Marthe) was obtained from Forschungsbrauerei Weihenstephan, and wheat (not for malting) was purchased from local retail. Malt samples, namely, pilsner malt, pale ale malt, Munich malt (Barke Munich malt), and red malt (Best RED-X) were purchased from a brewing supply (Hopfen and Mehr GmbH, Neukirch, Germany).

### Malting and Brewing Procedure

Sample beers were brewed in duplicate with a pilot-scale (80 L) brewing plant at the Forschungsbrauerei Weihenstephan, where several intermediate stages were collected during the brewing process (A*–F*, indicated in brackets). Therefore, malt samples were malted as standard according to MEBAK R-110.00.008, and standard malt parameters were analyzed based on the congress mash laboratory mashing regime R-206.00.002.^23^ The barley malt raw material (variety Cervinia, Saatzucht Breun, Herzogenaurach, Germany) was milled on a two-roller mill with a gap of 0.8 mm. Mashing was done using a standard mashing procedure, and the following steps were performed: 30 min at 62 °C, 30 min at 72 °C, and 10 min at 76 °C with a heating rate of 1 °C/min between the rests. After the mashing procedure, the mash was transferred to a lauter-tun preheated to 78 °C. After a 10 min rest in the lauter-tun, turbid first runnings were collected until clear and added back to the lauter-tun. The first runs were collected (sweet wort), and after the following sparge, the wort was collected in the kettle (collecting sample A*). After reaching a full wort-kettle, the wort was boiled for 60 min with the addition of hop pellet type 45 (variety Perle) to achieve 20 BU and a reference brew without hop dosage. After boiling, the wort was transferred to the whirlpool, where rest was held for 15 min prior to cooling down (collecting hopped sample B^+^ and unhopped reference B^–^). Fermentation was performed at 12 °C (yeast: Fermentis W34/70, Lambersart, France). Once the extract dropped below 3.5 °P (sampling C^+^/C^–^), fermentation was continued at 16 °C until the sensory threshold of diacetyl was reached and then cooled down to 0 °C (sampling D^+^/D^–^). Conditioning was carried out at 0 °C for 3 weeks, and the spunding pressure was set to give the beers a CO_2_ content of 4.0–4.5 g/L (sampling E^+^/E^–^). For filtration, Pall Seitz K 150 filter sheets (Pall Food and Beverage, New York) yielding a haze-free beer were used (sampling F^+^/F^–^). All beers were filled under CO_2_ conditions in 0.33 L long-neck bottles and stored at 4 °C. All standard wet-chemical wort and beer analyses were done according to MEBAK.^24^

### Solvent Extraction of Barley

Malting barley samples (500 g) were ground in a laboratory blender (Analysenmühle A10, Ika Labortechnik, Germany), defatted with pentane (3 × 500 mL), and then extracted with a 2-propanol/water mixture (80:20, v/v, 5 × 500 mL) at room temperature during ultrasonication. After filtration (MN 6151/4, 150 mm), the combined supernatants were separated from the organic solvents in a vacuum at 40 °C, lyophilized for 48 h, and then stored at −20 °C until further use.

### Fractionation of the 2-Propanol/Water-Extract by Medium Pressure Liquid Chromatography (MPLC)

The 2-propanol/water-extract (2 g) was dissolved in methanol/water (30:70, v/v, 20 mL) and injected through a six-way injection valve on a Sepacore system (Büchi, Flawil, Switzerland), consisting of two C-605 pumps, a C-620 control unit, a C-660 fraction collector, and a C-635 ultraviolet (UV) detector. Separation was performed on a 460 × 16 mm^2^ glass column (Büchi, Flawil, Switzerland) filled with a 25–40 μm LiChroprep RP18 material (Merck KGaA, Darmstadt, Germany). Operating at a flow rate of 30 mL/min, the solvent system consisted of aqueous formic acid (0.1%, A) and methanol (B), and the following gradient was used: 0 min/5% B, 20 min/30% B, 30 min/100% B, 40 min/100% B. Prior to the next injection, the column was flushed to 5% B for 3 min and kept for 10 min. The absorption at 280 nm was obtained using Sepacore Control Chromatography Software, version 1.0 (Büchi, Flawil, Switzerland), and three fractions (M1–M3) were collected before separation from the solvent in vacuum at 40 °C and freeze-dried for 48 h.

### Isolation and Structure Determination of Antioxidant Precursors from MPLC Fraction M1

For further purification using HPLC, fraction M1 was dissolved in water and, after membrane filtration, it was injected onto a 250 × 21.2 mm^2^ inner diameter, 5 μm, Luna Phenyl-Hexyl column (Phenomenex, Aschaffenburg, Germany) with a flow rate of 21 mL/min using a 2 mL sample loop. Using a binary gradient of 0.1% aqueous formic acid (v/v) as solvent A and acetonitrile as solvent B, chromatography was performed using 2% B for 25 min. The effluent was monitored at 280 nm, and four subfractions, namely M1–1 up to M1–4, were individually collected in several runs; the corresponding fractions were combined. The fractions were separated from the solvent under vacuum at 40 °C, followed by lyophilization. Based on the comparison of both spectroscopic and spectrometric data (ultraviolet–visible (UV/vis), liquid chromatography-time-of-flight-mass spectrometry (LC-TOF-MS), ^1^H/^13^C NMR), the structures of the compounds previously reported from wheat germ were confirmed as 4-hydroxyphenyl-β-d-glucopyranosyl-(1 → 6)-β-d-glucopyranosyl-(1 → 6)-β-d-glucopyranoside (Arbutintrioside, **39**), 4-hydroxy-3-methoxyphenyl-β-d-glucopyranosyl-(1 → 6)-β-d-glucopyranosyl-(1 → 6)-β-d-glucopyranoside (Tachiotrioside, **40**), 4-hydroxy-2-methoxyphenyl-β-d-glucopyranosyl-(1 → 6)-β-d-glucopyranosyl-(1 → 6)-β-d-glucopyranoside (Isotachiotrioside, **40a**), and 4-hydroxy-3-methoxyphenyl-β-d-glucopyranosyl-(1 → 6)-β-d-glucopyranoside (Tachiodioside, **41**).^25^

### Isolation and Structure Determination of Antioxidant Precursors from MPLC Fraction M3

M3 was separated after dissolution in water by preparative HPLC on a 250 × 21.2 mm^2^ inner diameter, 5 μm, Luna Phenyl-Hexyl column (Phenomenex, Aschaffenburg, Germany) at a flow rate of 21 mL/min. Chromatography was performed by eluting with 15% B within 12 min using a solvent system consisting of 0.1% formic acid in water (solvent A) and acetonitrile (solvent B). Monitoring the effluent at 228 nm revealed that purified hordatine glucosides (**42**–**44**) were collected. For the separation of hordatine glucosides, an alternative method was applied, using a 250 × 10.0 mm^2^ i.d., 5 μm, Luna PFP(2) column (Phenomenex, Aschaffenburg, Germany) with 0.1% trifluoroacetic acid in water (v/v) as solvent A and 0.1% trifluoroacetic acid in methanol (v/v) as solvent B, and the absorption was detected at 280 nm. The following gradient was used for separation at a flow rate of 5.5 mL/min: 0 min/15% B, 10 min/30% B, 24 min/42% B, 27 min/100% B, 30 min/100% B, 32 min/15% B, and 35 min/15% B. After rechromatography and verifying the purity of each fraction using analytical HPLC, the components were separated from the organic solvent under vacuum at 40 °C, freeze-dried for 48 h, and then studied by LC–TOF–MS and one-dimensional/two-dimensional-nuclear magnetic resonance (1D/2D-NMR) spectroscopy. The structure of the previously reported hordatine A glucoside (**42**) was confirmed in accordance with the spectroscopic and spectrometric data,^12,26^ but a *cis-*configuration was found for the aliphatic double bond. The chemical structures of hordatine B glucoside (**43**) and hordatine C glucoside (**44**) were determined using UV/vis, LC-TOF-MS, and 1D/2D NMR experiments. The ^1^H- and ^13^C NMR data are shown in Table 1.

**Table 1:** ^1^H and ^13^C NMR Data (500/125 MHz, D_2_O with 5% methanol-*d*_*4*_) of Hordatine A Glucoside (**42**), Hordatine B Glucoside (**43**), and Hordatine C glucoside (**44**)

	hordatine A glucoside (**42**)				hordatine B glucoside (**43**)				hordatine C glucoside (**44**)			
position	δ_C_ [ppm]	HSQC	δ_H_ [ppm]	M (*J* [Hz])	δ_C_ [ppm]	HSQC	δ_H_ [ppm]	M (*J* [Hz])	δ_C_ [ppm]	HSQC	δ_H_ [ppm]	M (*J* [Hz])
C(1)	130.2	[C]			131.3	[C]			131.4	[C]		
C(2)	125.6	[CH]	7.28	s	118.1	[CH]	6.90	s	118.0	[CH]	6.90	s
C(3)	126.8	[C]			127.6	[C]			127.8	[C]		
C(4)	159.9	[C]			148.5	[C]			147.0	[C]		
C(5)	110.7	[CH]	6.99	d (8.4)	144.6	[C]			144.8	[C]		
C(6)	132.0	[CH]	7.36	d (8.4)	114.5	[CH]	7.09	d (8.4)	114.5	[CH]	7.10	s
C(7)	137.1	[CH]	6.89	d (12.3)	137.1	[CH]	6.87	d (12.3)	137.1	[CH]	6.89	d (12.2)
C(8)	123.5	[CH]	6.04	d (12.3)	124.0	[CH]	6.06	d (12.3)	124.2	[CH]	6.07	d (12.2)
C(9)	171.6	[C]			171.6	[C]			171.8	[C]		
C(10)					57.1	[CH_3_]	3.90	s	57.1	[CH_3_]	3.93	s
C(1′)	134.8	[C]			134.6	[C]			135.4	[C]		
C(2′)	128.6	[CH]	7.44	d (8.7)	128.6	[CH]	7.42	d (8.7)	111.4	[CH]	7.13	s
C(3′)	117.8	[CH]	7.20	d (8.7)	117.8	[CH]	7.18	d (8.7)	146.8	[C]		
C(4′)	158.0	[C]			158.0	[C]			150.1	[C]		
C(5′)	117.8	[CH]	7.20	d (8.7)	117.8	[CH]	7.18	d (8.7)	117.0	[CH]	7.23	d (8.2)
C(6′)	128.6	[CH]	7.44	d (8.7)	128.6	[CH]	7.42	d (8.7)	119.7	[CH]	7.04	d (8.2)
C(7′)	88.5	[CH]	5.94	d (7.2)	89.0	[CH]	5.97	d (7.1)	89.1	[CH]	5.98	d (7.0)
C(8′)	57.8	[CH]	4.32	d (7.2)	58.1	[CH]	4.33	d (7.1)	58.3	[CH]	4.34	d (7.0)
C(9′)	174.0	[C]			173.8	[C]			173.9	[C]		
C(10′)									57.1	[CH_3_]	3.89	s
C(1″)	40.0	[CH_2_]	3.03–3.35	m	40.0	[CH_2_]	3.01–3.36	m	40.0	[CH_2_]	2.98–3.27	m
C(1‴)	39.7	[CH_2_]	3.03–3.35	m	39.7	[CH_2_]	3.01–3.36	m	39.8	[CH_2_]	2.98–3.27	m
C(2″), C(2‴), C(3″), C(3‴)	26.1, 26.2, 26.3, 26.6	[CH_2_]	1.34–1.61	m	26.2, 26.3, 26.6, 26.6	[CH_2_]	1.34–1.62	m	26.2, 26.3, 26.7, 26.7	[CH_2_]	1.33–1.65	m
C(4″), C(4‴)	41.7	[CH_2_]	3.03–3.35	m	41.7	[CH_2_]	3.01–3.36	m	41.7	[CH_2_]	2.98–3.27	m
C(5″), C(5‴)	157.6	[C]			157.6	[C]			157.8	[C]		
C(1″″)	100.9	[CH]	5.17	d (7.3)	100.9	[CH]	5.16	d (7.2)	101.2	[CH]	5.17	d (7.0)
C(2″″)	73.8	[CH]	3.58–3.71	m	73.8	[CH]	3.58–3.70	m	73.8	[CH]	3.60–3.70	m
C(3″″)	76.5	[CH]	3.58–3.71	m	76.5	[CH]	3.58–3.70	m	76.5	[CH]	3.60–3.70	m
C(4″″)	70.4	[CH]	3.52	pt (9.0)	70.4	[CH]	3.52	pt (9.0)	70.4	[CH]	3.54	pt (9.0)
C(5″″)	77.1	[CH]	3.58–3.71	m	77.1	[CH]	3.58–3.70	m	77.1	[CH]	3.60–3.70	m
C(6″″)	61.5	[CH_2_]	3.78	dd (2.0, 12.4)	61.5	[CH_2_]	3.77	dd (2.0, 12.4)	61.5	[CH_2_]	3.78	dd (5.8, 12.2)
			3.96	dd (5.8, 12.4)			3.95	dd (5.8, 12.4)			3.94	dd (1.8, 12.2)

#### Hordatine A Glucoside (**42**)

UV/vis (0.1% aqueous formic acid/acetonitrile, 65:35, v/v): λ_max_ = 228/300/320 nm; LC-TOF-MS (ESI)^−^: *m*/*z* (%) 757.3530 (100; measured), 757.3521 (calculated for [C_34_H_48_N_8_O_9_ + HCOOH – H]^−^), 711.3482 (5; measured), 711.3466 (calculated for [C_34_H_48_N_8_O_9_ – H]^−^); LC-TOF-MS (ESI)^+^: *m*/*z* (%) 713.3620 (10; measured), 713.3623 (calculated for [C_34_H_48_N_8_O_9_ + H]^+^), 357.1849 (100; measured), 357.1851 (calculated for [C_34_H_48_N_8_O_9_ + 2H]^2+^).

#### Hordatine B Glucoside (**43**)

UV/vis (0.1% aqueous formic acid/acetonitrile, 65:35, v/v): λ_max_ = 228/300/320 nm; LC-TOF-MS (ESI)^−^: *m*/*z* (%) 787.3631 (100; measured), 787.3626 (calculated for [C_35_H_50_N_8_O_10_ + HCOOH – H]^−^), 741.3563 (5; measured), 741.3572 (calculated for [C_35_H_50_N_8_O_10_ – H]^−^); LC-TOF-MS (ESI)^+^: *m*/*z* (%) 743.3723 (5; measured), 743.33728 (calculated for [C_35_H_50_N_8_O_10_ + H]^+^), 372.1902 (100; measured), 372.1904 (calculated for [C_35_H_50_N_8_O_10_ + 2H]^2+^).

#### Hordatine C Glucoside (**44**)

UV/vis (0.1% aqueous formic acid/acetonitrile, 65:35, v/v): λ_max_ = 228/300/320 nm; LC-TOF-MS (ESI)^−^: *m*/*z* (%) 817.3743 (100; measured), 817.3732 (calculated for [C_36_H_52_N_8_O_11_ + HCOOH – H]^−^), 771.3676 (5; measured), 771.3677 (calculated for [C_36_H_52_N_8_O_11_ – H]^−^); LC-TOF-MS (ESI)^+^: *m*/*z* (%) 773.3829 (10; measured), 773.3834 (calculated for [C_36_H_52_N_8_O_11_ + H]^+^), 387.1956 (100; measured), 387.1957 (calculated for [C_36_H_52_N_8_O_11_ + 2H]^2+^).

### Quantitative Analysis of Antioxidants and Antioxidant Precursors

The quantitation by high-performance liquid chromatography-triple quadrupole mass spectrometry (HPLC-MS/MS), operating in multiple reaction monitoring (MRM) modes and leveraging both internal standards and the ECHO technique, followed a protocol published very recently.^27^

### Sample Preparation

Wort and beer samples from the brewing process were degassed for 10 min upon ultrasonification and membrane filtration and directly investigated by HPLC-MS/MS. Wheat and malting barley raw material and barley malt types were ground in a laboratory blender (Analysenmühle A10, Ika Labortechnik, Germany), and aliquots (2.5 g) were extracted with methanol/water (70:30, v/v; 3 × 10 mL) by ultrasonification. After centrifugation (4000 rpm, 2 min), the organic solvent in the combined supernatants was removed with a nitrogen stream at room temperature, and the solution was lyophilized for 48 h. The extract was redissolved in methanol/water (70/30, v/v; 10 mL) and analyzed by HPLC-MS/MS after membrane filtration.

### Yeast Metabolism Model Experiments

Yeast-nitrogen base without amino acids (700 mg) and glucose (10 g) was dissolved in phosphate buffer (100 mM, pH 5.2; 100 mL). An aliquot (10 mL) was mixed with dry yeast (30 mg, commercial dry baker’s yeast) (II), tachiotrioside (**40**) (2930 mg/L in water, 20 μL) (III), or both dry yeast (30 mg) and tachiotrioside (**40**) (2930 mg/L in water, 20 μL) (I). Samples were collected at the beginning, after 1 day, and after 2 days, and after membrane filtration, they were directly analyzed by HPLC-MS/MS.

### Recovery Experiments for Malt as Matrix

After the concentrations of the antioxidants in the pilsner-type malt (control) were determined, the sample was spiked (in triplicate) with tyrosine (**33**) (1496 μmol/kg), tryptophan (**32**) (840 μmol/kg), catechin (**20**) (23 μmol/kg), and ferulic acid (**6**) (44 μmol/kg). After sample workup as reported above, the quantitation by HPLC-MS/MS revealed the following recovery rates: tyrosine (**33**) 85.3%, tryptophan (**32**) 96.0%, catechin (**20**) 119.0%, and ferulic acid (**6**) 113.5%.

### Data Visualization

Data analysis and visualization were performed within the programming environment R (version 3.5.2), whereby the heatmaps were plotted using heatmap.2 function of the gplots package based on the raw concentration data (Tables S1 and S2 of the Supporting Information) after autoscaling or log-transformation.

### Estimation of the Antioxidant Activity In Vitro

The antioxidant capability of the purified compounds was measured by applying three in vitro assays, namely oxygen radical absorbance capacity (ORAC) assay, hydrogen peroxide scavenging assay, and linoleic acid assay, following a previously described protocol.^10^

### High-Performance Liquid Chromatography (HPLC)

The HPLC system (Jasco, Groß-Umstadt, Germany) consisted of two PU-2087 Plus pumps, a DG-2080-53 degasser, and an MD-2010 Plus diode array detector to monitor the effluent in a range between 220 and 500 nm using Chrompass 1.8.6.1 software (Jasco, Groß-Umstadt, Germany). For sample injection, an AS-2055 Plus autosampler was used in analytical mode and a 7725i type Rheodyne injection valve (Rheodyne, Bensheim, Germany) in preparative and semipreparative modes.

### UPLC/Time-of-Flight Mass Spectrometry (UPLC-TOF-MS)

Aliquots (2 μL) of all antioxidants were injected into an Acquity UPLC core system (Waters, Manchester, U.K.), consisting of a binary solvent manager, a sample manager, and a column oven. Chromatographic separation was performed on a 150 × 2 mm^2^ i.d., 1.7 μm, BEH C18 column (Waters, Manchester, U.K.) at a flow rate of 0.4 mL/min and a temperature of 40 °C. Aqueous formic acid (0.1%, A) and acetonitrile (B) were used as solvents for the following gradient: 0 min/5% B, 3 min/100% B, and 4 min/100% B. High-resolution mass spectra were recorded on a Synapt G2-S HDMS (Waters, Manchester, U.K.) in negative and positive ESI resolution modes using −3.0 and +2.5 kV capillary voltages, respectively, 30 kV sampling cone, 4.0 kV extraction cone, 150 °C source temperature, 450 °C desolvation temperature, 30 L/h cone gas, and 850 L/h desolvation gas. The instrument was calibrated (*m*/*z* 50–1200) using a solution of sodium formate (0.5 mM) dissolved in 2-propanol/water (9:1, v/v). All data were lock-mass-corrected using leucine enkephalin as a reference (*m*/*z* 554.2615, [M – H]^−^ and *m*/*z* 556.2771, [M + H]^+^). Data acquisition and interpretation were performed using MassLynx (version 4.1) and the “elemental composition” tool as software.

### Nuclear Magnetic Resonance (NMR) Spectroscopy

One- and two-dimensional ^1^H and ^13^C NMR spectra were recorded on a 400 MHz ultra shield Avance III spectrometer with a Broadband Observe BBFOplus probe head, and a 500 MHz ultra shield plus Avance III spectrometer with a Triple Resonance Cryo Probe TCI probe head (Bruker, Rheinstetten, Germany), respectively. Using methanol-*d*_4_ and D_2_O as solvents, the chemical shifts are expressed in parts per million relative to the solvent signal. When the compounds were analyzed in D_2_O, 20 μL of methanol-*d*_4_ was added for ^13^C-referencing. The pulse sequences for recording 2D NMR experiments (i.e., COSY, HSQC, HMBC, and *J*_res_) were obtained from the Bruker software library as taken from the literature for 1,1-ADEQUATE.^28^ Data processing was performed using XWin-NMR version 3.5 (Bruker, Rheinstetten, Germany) and Mestre-Nova 8 (Mestrelab Research, Santiago de Compostela, Spain) software.

## Results and Discussion

**Figure 1:**
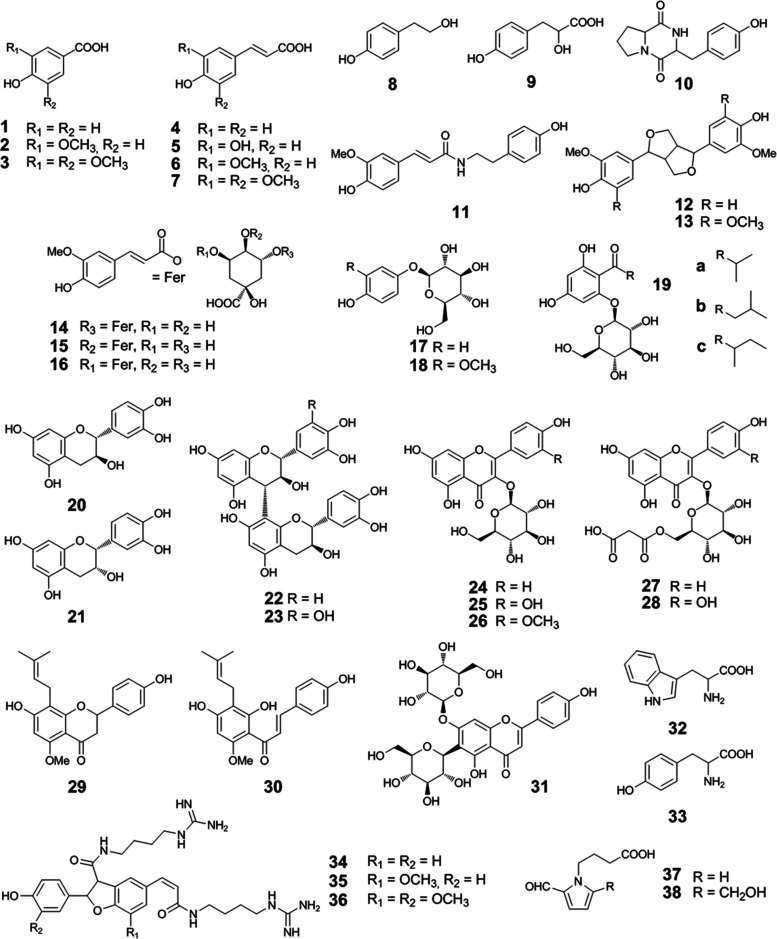
Chemical structures of antioxidants identified in beer: *p*-hydroxybenzoic acid (**1**), vanillic acid (**2**), syringic acid (**3**), *p*-coumaric acid (**4**), caffeic acid (**5**), ferulic acid (**6**), sinapic acid (**7**), tyrosol (**8**), *p*-hydroxyphenyllactic acid (**9**), *cyclo* (Pro-Tyr) (**10**), *N-*feruloyltyramine (**11**), pinoresinol (**12**), syringaresinol (**13**), 5-feruloylquinic acid (**14**), 4-feruloylquinic acid (**15**), 3-feruloylquinic acid (**16**), arbutin (**17**), tachioside (**18**), *co*-multifidol glucoside (**19a**), *n*-multifidol glucoside (**19b**), *ad*-multifidol glucoside (**19c**), (+)-catechin (**20**), (−)-epicatechin (**21**), procyanidin B_3_ (**22**), prodelphinidin B_3_ (**23**), kaempferol glucoside (**24**), quercetin glucoside (**25**), isorhamnetin glucoside (**26**), kaempferol malonylglucoside (**27**), quercetin malonylglucoside (**28**), isoxanthohumole (**29**), xanthohumole (**30**), saponarin (**31**), tryptophan (**32**), tyrosine (**33**), hordatine A (**34**), hordatine B (**35**), hordatine C (**36**), 4-(2-formylpyrrol-1-yl)butyric acid (**37**), and 4-[2-formyl-5-(hydroxymethyl)pyrrol-1-yl]butyric acid (**38**).

In order to develop strategies to increase the yield of antioxidants during the beer brewing process and, consequently, improve flavor stability, initial data should be obtained about the origin and evolution of beer antioxidants. To monitor the conceivable formation or degradation of the antioxidants compared to an unhopped control (F^–^) and to highlight the steps with the highest modulating potential, wort and beer samples were collected at six different points during the brewing process, ranging from sweet wort (A*) to final beer (F^+^) (Figure 1).

### Concentration of Antioxidants in Wort and Beer Samples

**Figure 2:**
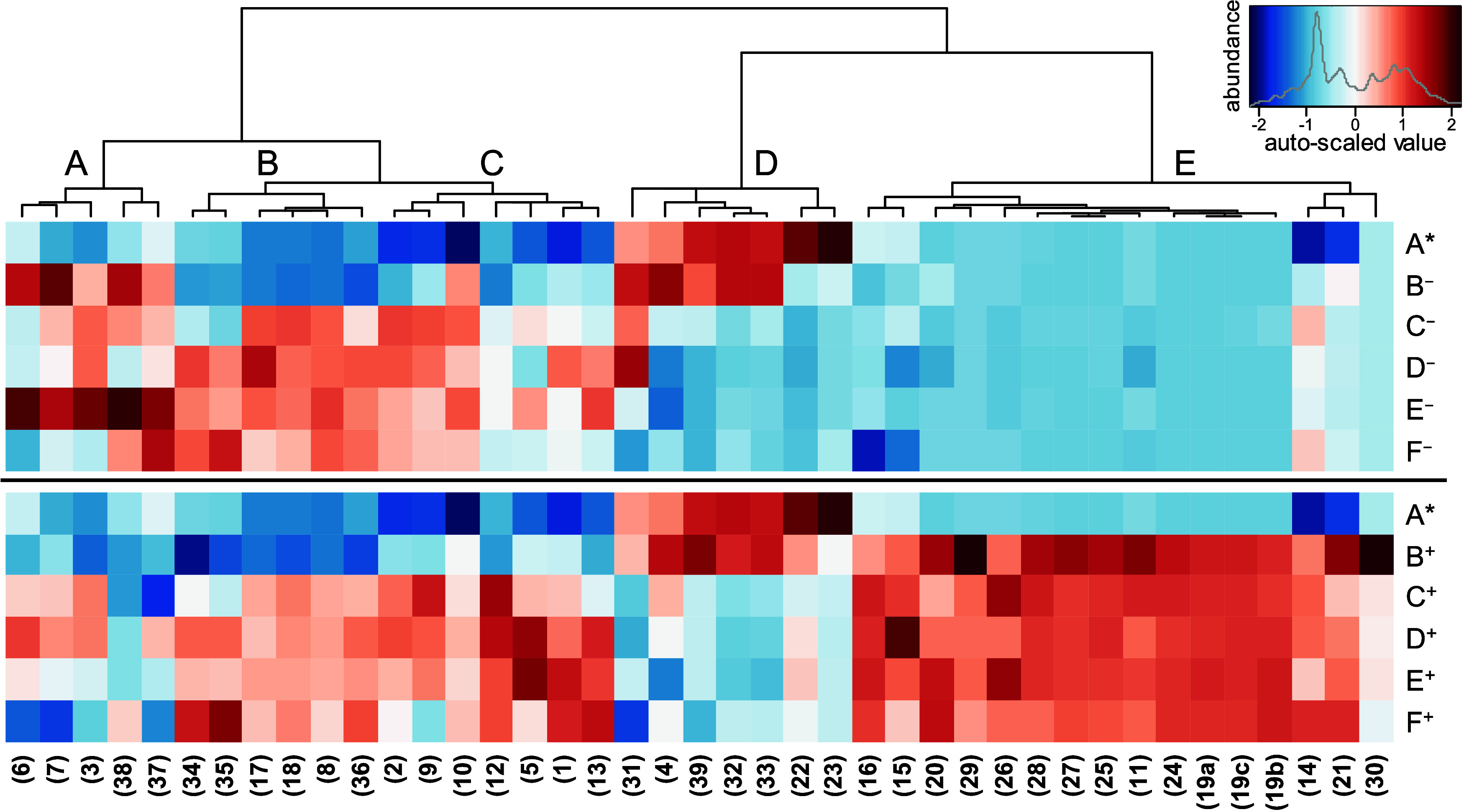
Heatmap with hierarchical cluster analysis of the mean-centered and unit variance-scaled concentrations of beer antioxidants in unhopped and hopped wort and beer samples (A*–F*; in μmol/L), with the structures shown in Figure 1.

**Figure 3:**
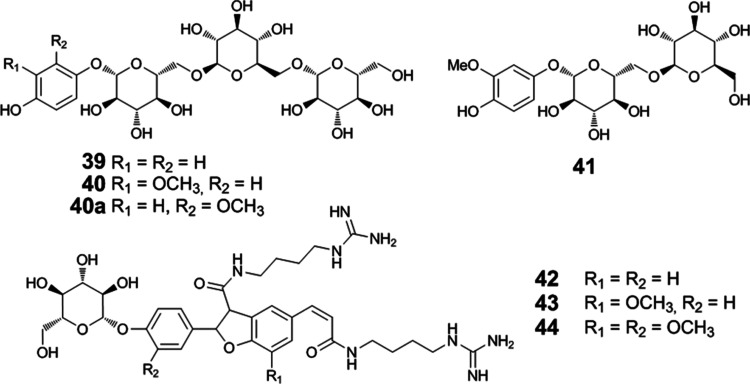
Chemical structures of antioxidant precursors isolated from barley, such as 4-hydroxyphenyl-β-d-glucopyranosyl-(1 → 6)-β-d-glucopyranosyl-(1 → 6)-β-d-glucopyranoside (arbutintrioside, **39**), 4-hydroxy-3-methoxyphenyl-β-d-glucopyranosyl-(1 → 6)-β-d-glucopyranosyl-(1 → 6)-β-d-glucopyranoside (tachiotrioside, **40**), 4-hydroxy-2-methoxyphenyl-β-d-glucopyranosyl-(1 → 6)-β-d-glucopyranosyl-(1 → 6)-β-d-glucopyranoside (isotachiotrioside, **40a**), 4-hydroxy-3-methoxyphenyl-β-d-glucopyranosyl-(1 → 6)-β-d-glucopyranoside (tachiodioside, **41**), hordatine A glucoside (**42**), hordatine B glucoside (**43**), and hordatine C glucoside (**44**).

After analysis of the samples by LC-MS/MS_MRM_,^27^ first a hierarchical cluster analysis was performed to derive the different groups of antioxidants with similar behavior throughout the brewing process (Figure 2). Therefore, cluster E contains hop-derived compounds since they are detectable only in hopped samples after wort boiling (B^+^–F^+^), such as the multifidol glucosides (**19a**–**c**), quercetin malonylglucoside (**28**), and xanthohumol (**30**). Exceptions within this cluster were 3- and 4-feruloylquinic acid (**15**–**16**), showing just slightly higher concentrations in hopped beer (F^+^) with 1.79 and 0.94 μmol/L as compared to 1.16 and 0.72 μmol/L in unhopped beer (F^–^), and catechin (**20**) with a 35% lower content of 5.96 μmol/L in unhopped beer (F^–^). In contrast, the antioxidants of cluster A originate from malt, as they already occur in sweet wort (A*) and their levels only differ negligibly between hopped and unhopped beer. These include 4-[2-formyl-5-(hydroxymethyl)pyrrol-1-yl]butyric acid (**38**) with 1.13 and 1.16 μmol/L, as well as saponarin (**31**) with 1.39 and 1.44 μmol/L, respectively. During manufacturing, just minor changes occurred. The same trend was observed for the compounds in cluster C, which were largely stable although they increased notably during wort boiling. This might be due to thermal processes, as in the case of *cyclo*(l-Pro-l-Tyr) (**10**), which increased from 1.02 in sweet wort (A*) to 1.96 and 2.25 μmol/L in both hopped (B^+^) and unhopped wort (B^–^), as also discussed for other diketopiperazines.^29^ Compounds in cluster D, however, clearly decrease in the brewing process. The concentrations of procyanidin B_3_ (**22**) and prodelphinidin B_3_ (**23**) dropped already during wort boiling from 18.80 and 12.92 μmol/L in sweet wort (A*) to 12.75 and 5.70 μmol/L in hopped wort (B^+^), and 7.75 and 5.04 μmol/L in unhopped wort (B^–^), respectively. This corresponds to a degradation of 55–60%, which might be linked to the formation of haze particles,^30^ and suggests, furthermore, an import of procyanidin B_3_ (**22**) from hops, as already described.^16^ Tyrosine (**33**) and tryptophan (**32**), also occurring in cluster D, degraded predominantly during fermentation by about 35%, as in the case of hopped samples with contents of 380 tyrosine (**33**) and 203 μmol/L tryptophan (**32**) in wort (B^+^) and 235 and 135 μmol/L, respectively, after fermentation (C^+^). For tyrosine (**33**), a conversion to tyrosol (**8**) is well known and can be observed by the reverse pattern,^31^ revealing an increase during fermentation from just 0.38 (B^+^) and 0.35 μmol/L (B^–^) in the wort samples to 37.1 (C^+^) and 45.1 μmol/L (C^–^) after fermentation. Similarly, the compounds in cluster B behaved independently of hopping during the brewing process. Though tachioside (**18**) and arbutin (**17**) already occurred in the wort samples (B*) with 14 and 0.85 μmol/L, their contents increased to levels of 35 and 1.5 μmol/L (C*), and did not significantly change further during warm and cold maturation, leading to concentrations of 33 and 1.4 μmol/L in beer (F*). Furthermore, hordatines (**34**–**36**), appearing in the same cluster, steadily increased throughout the beer production, such as in the case of hordatine B (**35**) from a level of 2.2 in wort (B*), 3.1 (C*), and 4.6 μmol/L (D*) after fermentation and warm maturation, up to 5.4 μmol/L in beer (F*). However, it is not clear how these important antioxidants are generated or released. In the literature, hordatine glucosides are known from barley besides hordatines (**34**–**36**),^11^ and from wheat germ tachioside (**18**), as well as corresponding oligosaccharides, have been reported (Figure 3).^25,32^ However, there is no information about such compounds in beer or that yeast is possibly able to metabolize them. Hence, in order to bridge this knowledge gap, barley was investigated as the precursor of the key antioxidants.

### Fractionation of Barley

**Figure 4:**
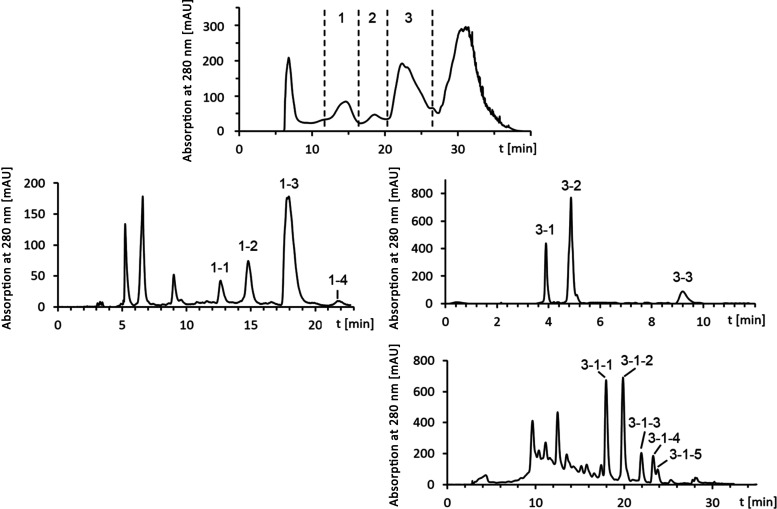
HPLC chromatograms of the fractionation to isolate antioxidant precursors **39**–**44** from barley.

To track down potential phenol glucosides, barley was extracted with 2-propanol/water (80/20, v/v), based on a literature protocol for wheat flour.^33^ After fractionation by MPLC, investigation by LC-TOF-MS revealed the target compounds in fractions M1 and M3, which were further subfractionated by preparative HPLC (Figure 4).

### Isolation and Structure Determination of Di- and Triglucosides (**39**–**41**) in M1

**Figure 5:**
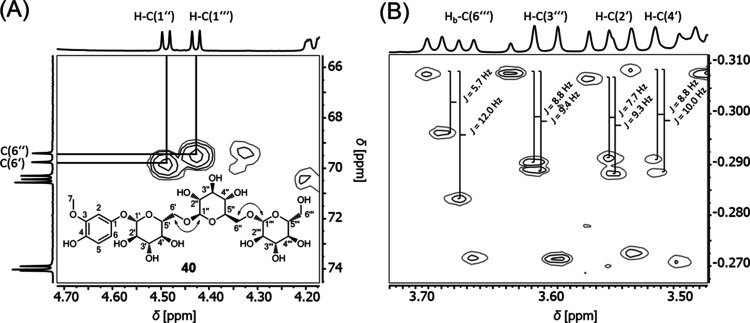
Excerpt of (**A**) the HMBC NMR spectrum (500/125 MHz, D_2_O with 5% methanol-*d*_4_), and (**B**) the *J*-resolved spectrum (500 MHz, D_2_O with 5% methanol-*d*_4_) of tachiotrioside (**40**).

**Figure 6:**
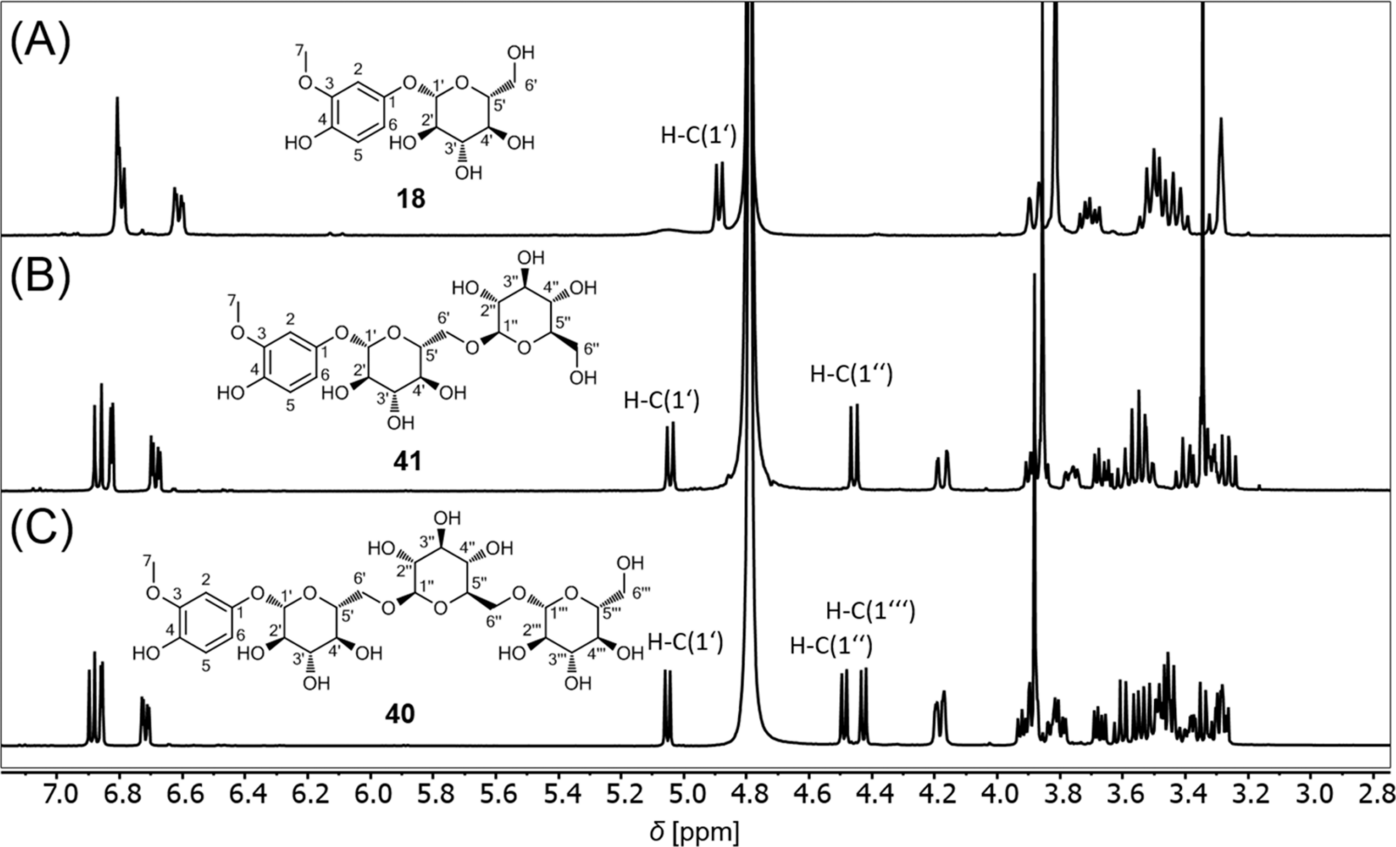
^1^H NMR spectra (500 MHz, D_2_O with 5% methanol-d_4_) of (**A**) tachioside (**18**), (**B**) tachiodioside (**41**), and (**C**) tachiotrioside (**40**).

After preparative isolation, 4-hydroxyphenyl-β-d-glucopyranosyl-(1 → 6)-β-d-glucopyranosyl-(1 → 6)-β-d-glucopyranoside (arbutintrioside, **39**) was identified in fraction M1-1, 4-hydroxy-3-methoxyphenyl-β-d-glucopyranosyl-(1 → 6)-β-d-glucopyranoside (tachiodioside, **41**) in fraction M1-2, the predominant 4-hydroxy-3-methoxyphenyl-β-d-glucopyranosyl-(1 → 6)-β-d-glucopyranosyl-(1 → 6)-β-d-glucopyranoside (tachiotrioside, **40**) in fraction M1-3, and 4-hydroxy-2-methoxyphenyl-β-d-glucopyranosyl-(1 → 6)-β-d-glucopyranosyl-(1 → 6)-β-d-glucopyranoside (isotachiotrioside, **40a**) in fraction M1-4 (Figure 3). The identification was achieved through the determined spectroscopic and spectrometric data (UV/vis, LC-TOF-MS, ^1^H/^13^C NMR) in comparison with the literature on wheat germ.^25^ As none of the glycoside structures were known from barley, the structural key elements were confirmed by additional 2D-NMR experiments (COSY, HSQC, HMBC). For tachiotrioside (**40**), unambiguous assignment of all signals was achieved by performing further a *J*-resolved and 1,1-ADEQUATE-experiment (Figure 5). As an example, the β-1 → 6-linkages of the sugar moieties of **40** was confirmed via the HMBC spectrum, revealing a ^3^*J* coupling between the protons at 4.43 ppm (H–C(1‴)) and 4.49 ppm (H–C(1″)) with the carbon atoms at positions C-6′ and C-6″(69.8 and 69.4 ppm). Using the *J*-resolved spectrum, all coupling constants were highlighted, which led to the recognition of three glucose moieties, based on the pattern of coupling constants, as reported in the literature.^34^ Moreover, the structural similarities of tachiotrioside (**40**) and tachiodioside (**41**) compared to tachioside (**18**) could be traced based on the ^1^H NMR spectra, exhibiting comparable aromatic signals, as the different number of anomeric proton signals described the number of glucose moieties (Figure 6).

### Isolation and Structure Determination of Hordatine Glucosides (**42**–**44**) in Fraction M3-1

After M3 was separated by preparative HPLC, another purification step by semipreparative HPLC was necessary to isolate pure compounds from fraction M3-1. Determination by UV/vis, LC-TOF-MS, and 1D/2D-NMR-experiments, respectively, revealed the structures of hordatine A glucoside (**42**) in subfractions M3-1-2 and M3-1-4, hordatine B glucoside (**43**) in subfractions M3-1-1 and M3-1-3, and hordatine C glucoside in subfraction M3-1-5 (**44**). In contrast to the literature,^12^ only the *cis*-isomers could be isolated and characterized, possibly due to a continuous *trans-* to *cis*-isomerization during fractionation, as previously described.^26^ First hints on the structures were obtained by UV/vis spectroscopy, with the subfractions of M3-1 exhibiting typical absorption maxima for hordatines and other hydroxycinnamic acid derivatives.^10^ In the following, the LC-TOF-MS analysis revealed the pseudomolecular ions ([M + H]^+^) at *m*/*z* 713.3620, *m*/*z* 743.3723, and *m*/*z* 773.3829, fitting well with the molecular formulas of C_34_H_48_N_8_O_9_, C_35_H_50_N_8_O_10_, and C_36_H_52_N_8_O_11_, respectively. An increase of methoxy functions was revealed by a mass shift of 30 Da, showing a pattern as already published.^35^ Furthermore, in relation to the corresponding hordatines (**34**–**36**),^10^ a mass shift of 162 Da in the MS spectra indicated modification with a hexose moiety. As described for hordatines (**34**–**36**),^10^ the [M+2H]^2+^ ions also appeared for the glucosides with the highest relative abundance, recorded at *m*/*z* 357.1849, *m*/*z* 372.1904, and *m*/*z* 387.1956. The application of 1D/2D-NMR experiments (Table 1) led to the *cis-*configuration of the double bond, highlighted by the coupling constant of 12.3 Hz between protons H–C(7) and H–C(8). Further structural elements also showed the same NMR pattern as the aglycones (**34**–**36**), such as the ^3^*J* proton coupling constant of 7.1 Hz between H–C(7′) and H–C(8′), suggesting an opposing steric arrangement of the amide and hydroxyphenyl side chains. By leveraging the HMBC NMR spectrum of hordatine B and C glucoside (**43** and **44**), the ^3^*J* correlation of H_3_–C(10) or H_3_–C(10′) and C(5) or C(3′) confirmed the aromatic substitution pattern. In the ^1^H NMR spectra of hordatine glucosides (**42**–**44**), further signals, caused by the hexose, were detectable in the range between 3.4 and 3.9 ppm (H–C(2″″), H–C(3″″), H–C(4″″), H–C(5″″), H_2_–C(6″″)), as well as the anomeric proton (H–C(1″″)) at 5.17 ppm. The homonuclear ^3^*J* coupling constants of the anomeric signals, ranging from 7.0 to 7.3 Hz for the three homologues, established the described β-d-glucosides.^11^

Nevertheless, no detailed ^1^H/^13^C NMR data were available, except for hordatine A glucoside (**42**),^12^ although hordatine B glucoside (**43**) has also been reported in barley.^11,36^ The chemical structure of hordatine C glucoside (**44**), to the best of our knowledge, has not been reported earlier, except for being suggested in barley based on MS data.^33^

### Concentrations of Precursors in Wort and Beer Samples

**Figure 7:**
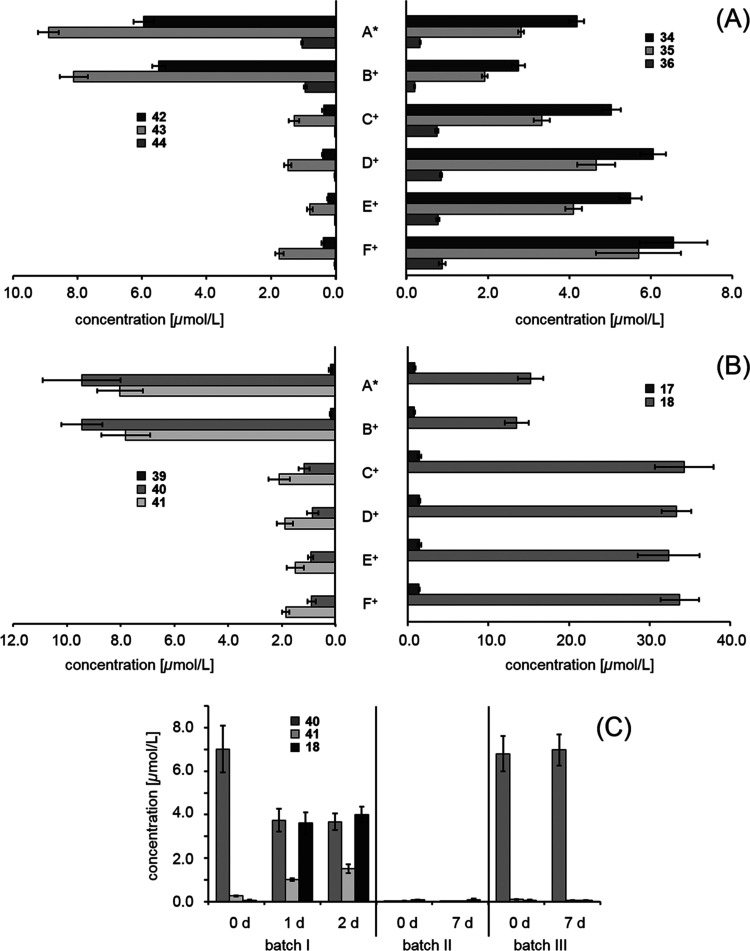
Evolution of the concentrations of antioxidants and their corresponding precursors throughout the brewing process (in μmol/L), plotting concentrations of (A) hordatine glucosides (**42**–**44**) against hordatines (**34**–**36**), (B) triglucosides **39**–**41** against monoglucosides **17**–**18**, and (C) tachiotrioside (**40**), tachiodioside (**41**), and tachioside (**18**) in the fermentation model. Chemical structures are shown in Figures 1 and 2.

After their structures were elucidated, the content of the antioxidant precursors was investigated by HPLC-MS/MS_MRM_, using samples from the different production stages, as described above (Figure 7). Since the precursors originated from barley, their concentrations were similar in hopped and unhopped samples. Nevertheless, an overwhelming degradation of 90% for tachiotrioside (**40**), 70% for tachiodioside (**41**), and >85% for hordatine glucosides (**42**–**44**) was determined during fermentation. For tachiotrioside (**40**) and tachiodioside (**41**), 9.44 and 7.81 μmol/L, respectively, were determined in the hopped wort (B^+^), as their contents declined to 1.17 and 2.10 μmol/L after fermentation (C^+^). The concentration of arbutintrioside (**39**) also decreased from 0.19 (B^+^) below the limit of quantitation of 0.07 μmol/L (C^+^). Isotachiotrioside (**40a**), however, was not detected in any sample. This agreed well with the trend observed for tachioside (**18**) and arbutin (**17**), as their levels increased during fermentation, as mentioned before. Hordatine glucosides (**42**–**44**) and aglycones (**34**–**36**) behaved in the same way. In the case of hordatine A glucoside (**42**), the concentration degraded nearly completely from 5.48 in the hopped wort (B^+^) to 0.36 μmol/L after fermentation (C^+^). In contrast, the content of aglycones (**34**–**36**) increased to a lesser extent and spread between fermentation and maturation. Hordatine glucosides (**42**–**44**) might be quickly absorbed from the medium by yeast, whereas aglycones (**34**–**36**) are slowly and incompletely released, possibly due to further metabolic transformation. Moreover, the concentration ratios of the three glucosides (**42**–**44**) and related aglycones (**34**–**36**) were different. As the highest amounts among the glucosides (**42**–**44**) were measured for hordatine B glucoside (**43**) with 8.90 in sweet wort (A*) compared to 5.95 μmol/L hordatine A glucoside (**42**) and 1.04 μmol/L hordatine C glucoside (**44**), hordatine A (**34**) was slightly dominating among aglycones (**42**–**44**). Even after degradation of the precursors, 6.56 μmol/L was recorded for hordatine A (**34**) in the final beer (F^+^), but only 5.70 μmol/L was recorded for hordatine B (**35**) and 0.88 μmol/L was recorded for hordatine C (**36**).

### Precursor Evolution in Fermentation Model Experiments

To confirm unequivocally that yeast is able to hydrolyze the β-1 → 6-linkages, to liberate tachioside (**18**) from tachiotrioside (**40**) and tachiodioside (**41**), a liquid fermentation model was developed. Therefore, a simple basic medium, consisting of a commercial yeast-nitrogen base without amino acids and glucose in a phosphate buffer at a typical wort pH value of 5.2, was used. In the first batch (I) (Figure 7C), **40** was added based on the measured concentrations in the wort samples as well as dry yeast. After 1 day of fermentation, the same pattern was observed as during wort fermentation: **40** declined from 7.01 to 3.74 μmol/L, and **41** and **18** could be quantified at levels of 1.01 and 3.61 μmol/L, respectively. Additionally, two control batches (II and III) were investigated: one batch without **40** (II) to consider a possible de novo formation of **18**, and another without dry yeast (III) to rule out the acid-catalyzed hydrolysis of **40**. As expected, neither **18**, **40**, nor **41** were found in the first control batch (II) even after 7 days of fermentation, while **40** remained constant in the second control batch (III). Hence, it was unambiguously shown that *Saccharomyces cerevisiae* was able to hydrolyze β-1 → 6-linkages in **40** and **41**.

### Evaluation of the Antioxidant Activity of Purified Antioxidant Precursors

The antioxidant precursors were analyzed for their antioxidant activity, using the same tests as described previously for the antioxidants found in beer,^10^ namely an oxygen radical absorbance capacity (ORAC) assay, a hydrogen peroxide scavenging (HPS) assay, and a linoleic acid (LA) assay. Therefore, hordatine glucosides (**42**–**44**) were clearly exhibited in all three in vitro assays (Table 2), and their antioxidant activities were lower than those of the corresponding aglycones (**34**–**36**). For hordatine B glucoside (**43**), 3.40 μmol TE/μmol by the ORAC assay, 1.17 μmol TE/μmol by the HPS assay, and 1.31 μmol TE/μmol by the LA assay were evaluated. For hordatine B (**35**), 12.58 μmol TE/μmol by the ORAC assay, 3.02 μmol TE/μmol by the HPS assay, and 3.19 μmol TE/μmol by the LA assay were measured. This observation can be explained by the sugar moiety modifying only the phenolic hydroxyl function, which makes the abstraction of a hydrogen atom to form a primary radical energetically more difficult as compared to hordatines.^37,38^ Tachiotrioside (**40**) and tachiodioside (**41**), however, showed a very similar antioxidant activity compared to tachioside (**18**); therefore, the additional sugar moieties, apparently, have a negligible impact on the antioxidative properties (Table 2).

**Table 2:** Antioxidant Activity of the Purified Compounds Isolated from Barley And Beer (in μmol TE/μmol) with the Structures Shown in Figure 1, Investigated by ORAC Assay, HPS Assay, and LA Assay, Respectively

analyte (**no**.)	ORAC assay^b^	HPS assay^c^	LA assay^c^
hordatine A (**34**)^a^	10.01 ± 0.97	1.97 ± 0.44	1.05 ± 0.13
hordatine B (**35**)^a^	12.58 ± 0.98	3.02 ± 0.53	3.19 ± 0.32
hordatine C (**36**)**^a^**	17.50 ± 0.77	4.10 ± 1.01	6.14 ± 1.28
hordatine A glucoside (**42**)	1.94 ± 0.16	0.59 ± 0.05	1.04 ± 0.22
hordatine B glucoside (**43**)	3.40 ± 0.26	1.17 ± 0.19	1.31 ± 0.04
hordatine C glucoside (**44**)	4.76 ± 0.24	0.46 ± 0.20	1.01 ± 0.12
arbutin (**17**)	3.61 ± 0.16	1.40 ± 0.13	0.39 ± 0.05
tachioside (**18**)^a^	2.62 ± 0.14	0.98 ± 0.16	1.77 ± 0.23
arbutintrioside (**39**)	2.95 ± 0.25	1.78 ± 0.13	0.89 ± 0.13
tachiodioside (**41**)	2.50 ± 0.13	0.72 ± 0.12	1.81 ± 0.20
tachiotrioside (**40**)	2.19 ± 0.22	0.71 ± 0.05	1.95 ± 0.18
isotachiotrioside (**40a**)	1.98 ± 0.36	0.87 ± 0.22	1.16 ± 0.07

a Values taken from^10^

b Errors represent the standard deviation of four replicates.

c Errors represent the confidence interval (α = 5%) of each of the three replicates.

### Concentration of Antioxidants and their Precursors in Cereal and Malt Samples

**Figure 8:**
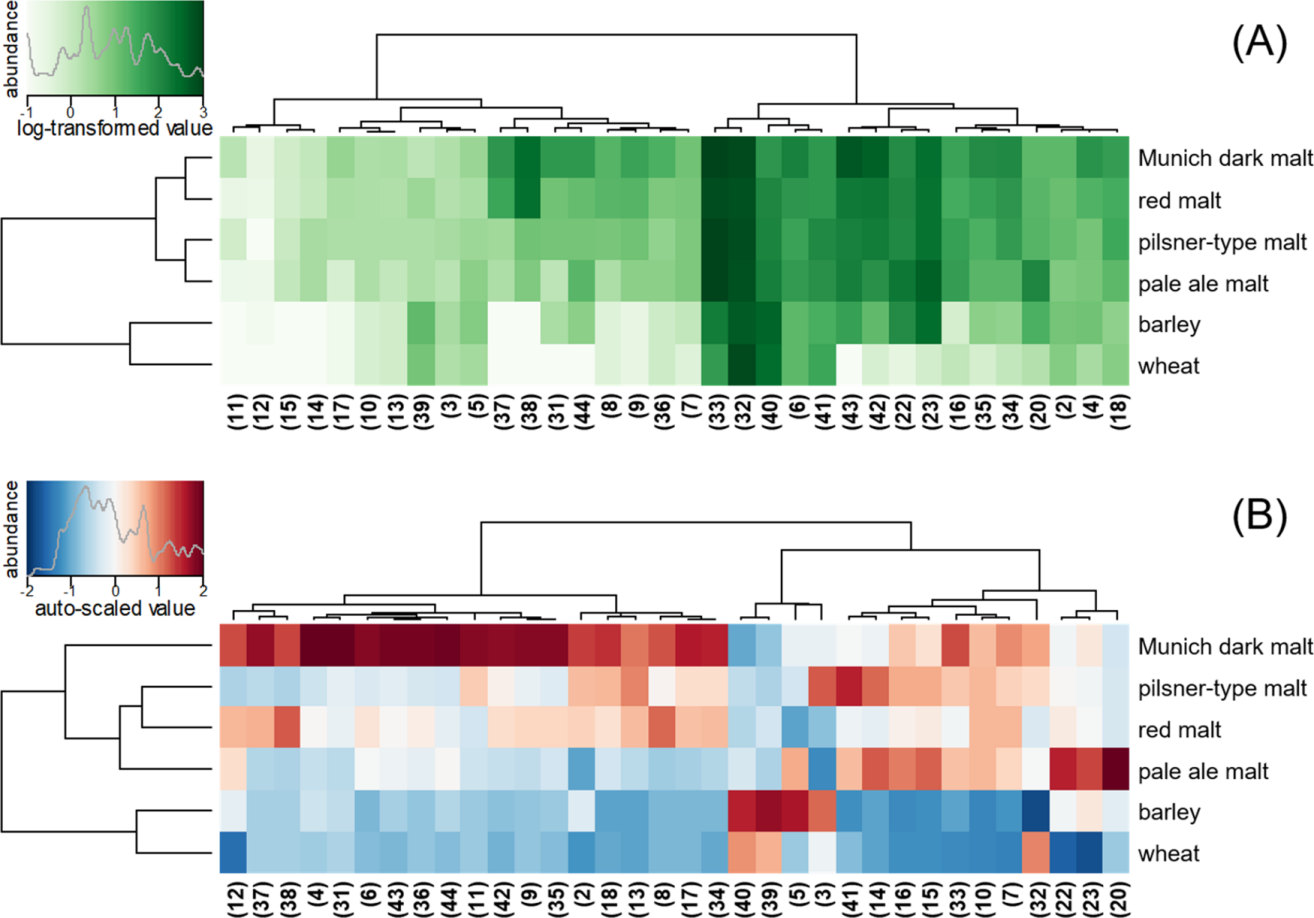
(A) Heatmap with hierarchical cluster analysis of the log_10_-transformed concentration of beer antioxidants in cereal and malt samples (in μmol/kg), and (B) the mean-centered and unit variance-scaled levels. Chemical structures are shown in Figures 1 and 2.

In order to gain insight into the modulating potential arising from the usage of different malt types, the natural concentration range of antioxidants and their precursors in the raw material was investigated. Therefore, besides a typical pilsner-type malt, a pale ale malt was investigated as well as a red and Munich malt, to cover a broad range of common malt, which can be added from a low dosage of 10–20% up to a rate of 100%. Moreover, barley and wheat were analyzed to determine the influence of the malting process on the yield of antioxidants and their precursors (Figure 8). The predominant compounds in the malt samples were, therefore, the amino acid tyrosine (**33**) at 700–1500 μmol/kg and tryptophan (**32**) at 650–780 μmol/kg. Nevertheless, prodelphinidin B_3_ (**23**) (240–430 μmol/kg) and procyanidin B_3_ (**22**) (100–200 μmol/kg) were also quantitatively dominant compounds, with the notably highest contents measured in pale ale malt. The levels of the identified antioxidant precursors were in the same range, namely tachiotrioside (**40**), from 63–140 μmol/kg in malt, hordatine A glucoside (**42**) from 78–430 μmol/kg, and hordatine B glucoside (**43**) from 130–560 μmol/kg (Figure 8A). The lowest concentration of tachiotrioside (**40**) was measured in Munich malt, and the amounts of hordatine glucosides (**42**–**44**) were more than two times higher than in any other investigated sample (Figure 8B). This demonstrates that the antioxidant content could significantly increase at a low dosage of such malt. The concentrations of aglycones (**34**–**36**) were also notably higher, as well as the contents of 4-[2-formyl-5-(hydroxymethyl)pyrrol-1-yl]butyric acid (**38**) with 284 μmol/kg in Munich and 267 μmol/kg in red malt, but just 10.6 and 7.1 μmol/kg in the investigated pilsner-type and pale ale malt, respectively. 4-[2-formyl-5-(hydroxymethyl)pyrrol-1-yl]butyric acid (**38**) was not detected in barley and wheat, which confirmed that the compound is an indicator of the degree of roasting formed during kilning. Overall, wheat showed a different composition, as neither hordatines (**34**–**36**), hordatine glucosides (**42**–**44**), nor saponarin (**31**), which are characteristic of barley, were detected. Tachiotrioside (**40**), however, at 297 μmol/kg is a major compound in wheat, as in barley at 374 μmol/kg, which is unequivocally above the level of the malt samples at 112 μmol/kg on average. Arbutintrioside (**39**) indicated the same tendency with 15 μmol/kg in barley, but a mean of 2.3 μmol/kg in malt, whereas tachiodioside (**41**) was found at a higher concentration in malt with 75.5 μmol/kg, as compared to 26.7 μmol/kg in barley. This assumes enzymatic depletion and suggests that arbutintrioside (**39**) and tachiotrioside (**40**) might be phytoanticipins, while arbutin (**17**) and tachioside (**18**) might be released in the case of oxidative stress.

Moreover, the contents of tyrosine (**33**) and tryptophan (**32**) obviously increased during malting, as 1070 and 710 μmol/kg occurred in malt, but 160 and 480 μmol/kg, respectively, were measured in barley. This might be linked to the degradation of proteins through enhanced enzymatic activity, and tryptophan (**32**) has already been described as an indicator of oxidative stress in barley leaves, as well as *p*-coumaroylagmatine, a biosynthetic precursor of hordatines (**34**–**35**).^39,40^ The amount of hordatines (**34**–**36**) and quantitatively dominant hordatine glucosides (**42**–**44**) also increased significantly during malting, like hordatine B glucoside (**43**) from 47 μmol/kg in barley to 260 μmol/kg in malt, or hordatine A (**34**) from 4.5 to 57 μmol/kg. Similar to the brewing process samples, hordatine B glucoside (**43**) tended to dominate among the glucosides (**42**–**44**), whereas the highest amounts were measured for hordatine A (**34**) among the aglycones (**34**–**36**). This might be due to enzymatic discrimination or different regulation mechanisms operating in parallel. Glucosides (**42**–**44**) and aglycones (**34**–**36**) might be phytoalexins, triggered by different stressors. So far, only hordatines (**34**–**36**) have been described as fungicides, with glucosides (**42**–**44**) showing a lower activity,^26,41^ correlating with their antioxidative properties.

In summary, large differences in the malt composition were revealed, which have a major effect on the antioxidant content of the resulting beer and can be leveraged to improve flavor stability. Significant modulation of the levels of antioxidants can be achieved using mixtures of diverse malt. Therefore, indications for characteristics of different malt varieties were gathered, although the number of analyzed samples was too low for statistically firm statements. It can be highlighted that the malting process has a crucial impact on the yield of antioxidants and their precursors, while wort boiling is particularly important for hop-derived compounds, and transformations during fermentation are critical for the antioxidant release. To confirm the observations of this investigation, quantitative investigations were carried out on commercial beer samples and were published separately.^27^

## Supporting Information Available

Detailed quantitative data of the investigated unhopped and hopped wort and beer samples and detailed quantitative data of the investigated wheat; barley; and malt samples (PDF)
