# Supplementary material for: Quantitative Antioxidant Profiling Throughout Beer Brewing Followed by Discovery and Isolation of Precursors from Barley (Hordeum vulgare L.)
Source: J Agric Food Chem. 2024 Jun 4;72(24):13885–97. doi: 10.1021/acs.jafc.4c00998 (PMC11191689; doi:10.1021/acs.jafc.4c00998)
Supplement: Supplementary file 1 — jf4c00998_si_001.pdf [file jf4c00998_si_001.pdf]

Supporting Information

Quantitative Antioxidant Profiling Throughout Beer Brewing Followed by Discovery and Isolation of Precursors from Barley (*Hordeum vulgare* L.)

Stefan Spreng[1], Julia Wannenmacher[2], Martina Gastl[2], Corinna Dawid[1,3], and Thomas Hofmann[1,3*]

[1]Chair of Food Chemistry and Molecular and Sensory Science, Technical University of Munich, Lise-Meitner-Str. 34, D-85354 Freising, Germany, [2]Chair of Brewing and Beverage Technology, Technical University of Munich, Weihenstephaner Steig 20, D-85354 Freising, Germany and [3]Bavarian Center for Biomolecular Mass Spectrometry, Gregor-Mendel-Straße 4, D-85354 Freising, Germany.

---

[*] **To whom correspondence should be addressed**

PHONE +49-8161/71-2902

FAX +49-8161/71-2949

E-MAIL thomas.hofmann@tum.de

Table S1: Concentration of Beer Antioxidants in Unhopped (Reference) and Hopped Wort and Beer Samples (A−F) in μmol/L.

analyte No.[a]	A*	B⁻	C⁻	D⁻	E⁻	F⁻	B⁺	C⁺	D⁺	E⁺	F⁺
**1**	0.81	1.32	1.48	1.79	1.47	1.45	1.38	1.61	1.76	1.96	1.92
**2**	1.63	2.14	3.60	3.52	3.21	3.21	2.45	3.44	3.58	3.17	2.90
**3**	0.30	0.43	0.46	0.46	0.54	0.36	0.28	0.45	0.45	0.38	0.33
**4**	3.47	3.75	3.19	2.91	2.86	3.11	3.68	3.39	3.27	2.91	3.27
**5**	0.17	0.21	0.25	0.21	0.27	0.23	0.23	0.26	0.32	0.33	0.25
**6**	11.2	12.2	11.2	11.2	12.5	10.8	10.8	11.5	11.9	11.4	10.5
**7**	1.53	1.88	1.70	1.66	1.83	1.63	1.58	1.69	1.73	1.64	1.46
**8**	0.34	0.35	45.1	45.4	49.3	46.5	0.38	37.1	38.7	37.2	32.1
**9**	0.50	0.64	0.81	0.79	0.73	0.74	0.62	0.85	0.80	0.78	0.62
**10**	1.02	2.25	2.39	2.14	2.41	2.13	1.96	2.06	2.17	2.08	2.13
**11**	0.05	0.05	0.04	0.03	0.05	0.04	0.19	0.16	0.14	0.15	0.14
**12**	0.22	0.20	0.28	0.29	0.29	0.27	0.21	0.40	0.39	0.36	0.36
**13**	1.17	1.28	1.31	1.41	1.45	1.31	1.22	1.32	1.47	1.45	1.49
**14**	0.43	0.67	0.79	0.72	0.71	0.78	0.84	0.87	0.86	0.78	0.91
**15**	0.86	0.81	0.85	0.74	0.79	0.72	1.01	1.04	1.16	1.02	0.94
**16**	1.52	1.37	1.44	1.43	1.45	1.16	1.69	1.83	1.81	1.83	1.79
**17**	0.85	0.85	1.62	1.79	1.58	1.37	0.82	1.45	1.41	1.47	1.40
**18**	15.2	14.4	37.1	35.1	34.4	31.7	13.5	34.3	33.3	32.3	33.7
**19a**	< 0.005	< 0.005	< 0.02	< 0.02	< 0.02	< 0.005	9.72	9.14	9.03	9.43	9.03
**19c**	< 0.005	< 0.005	< 0.02	< 0.005	< 0.005	< 0.005	0.87	0.84	0.83	0.84	0.82
**19b**	< 0.005	< 0.005	< 0.02	< 0.005	< 0.005	< 0.005	0.22	0.21	0.22	0.23	0.23
**20**	5.75	6.40	5.72	5.42	5.97	5.96	9.73	7.96	8.52	9.41	9.49
**21**	1.36	3.03	2.63	2.67	2.64	2.77	4.67	3.32	3.68	3.83	4.13
**22**	18.8	7.75	5.09	4.94	5.44	5.55	12.8	9.14	10.7	11.5	9.67

**23**	12.9	5.04	3.49	3.47	3.53	4.23	5.70	4.81	4.52	4.65	4.75
**24**	< 0.01	< 0.01	< 0.01	< 0.01	< 0.01	< 0.01	0.70	0.64	0.60	0.61	0.61
**25**	< 0.02	< 0.02	< 0.02	< 0.02	< 0.02	< 0.02	0.91	0.76	0.78	0.75	0.69
**26**	< 0.002	< 0.002	< 0.002	< 0.002	< 0.002	< 0.002	0.02	0.03	0.02	0.03	0.02
**27**	< 0.01	< 0.01	< 0.02	< 0.01	< 0.01	< 0.01	0.54	0.41	0.41	0.41	0.39
**28**	< 0.02	< 0.01	< 0.02	< 0.01	< 0.02	< 0.01	0.85	0.76	0.71	0.71	0.59
**29**	< 0.01	0.01	0.07	0.05	0.07	0.03	16.9	8.78	8.76	8.87	7.52
**30**	< 0.03	< 0.01	< 0.03	< 0.03	< 0.03	< 0.03	5.63	0.92	0.88	0.96	0.61
**31**	1.62	1.70	1.64	1.72	1.54	1.44	1.60	1.47	1.45	1.53	1.39
**32**	212	211	133	128	133	137	203	135	128	125	145
**33**	377	384	243	217	223	218	380	235	217	204	247
**34**	4.19	3.72	4.57	6.25	5.89	6.14	2.74	5.03	6.05	5.50	6.56
**35**	2.81	2.43	2.88	4.46	4.29	5.18	1.92	3.32	4.66	4.10	5.70
**36**	0.33	0.23	0.66	0.86	0.81	0.83	0.20	0.74	0.84	0.77	0.88
**37**	0.37	0.40	0.39	0.38	0.44	0.43	0.34	0.31	0.39	0.36	0.33
**38**	1.06	1.24	1.16	1.08	1.29	1.16	1.01	1.01	1.05	1.05	1.13

[a] Chemical structures are given in Figure 1

Table S2. Concentration of beer antioxidants and precursors in wheat, barley, and different barley malt types in *µ*mol/kg.

analyte No.[a]	wheat	barley	pilsner-type malt	Munich malt	red malt	pale ale malt
**2**	6.46	9.98	13.2	16.1	12.7	6.94
**3**	2.08	2.66	2.69	2.06	1.76	1.55
**4**	2.70	10.4	9.94	74.3	22.1	8.94
**5**	2.93	6.85	3.51	3.90	2.36	5.37
**6**	16.6	16.2	42.3	134	66.4	54.2
**7**	0.42	1.10	8.13	10.2	8.98	8.01
**8**	< 0.41	< 0.41	8.84	19.3	18.0	2.23
**9**	< 0.28	< 0.28	10.7	43.2	19.9	5.35
**10**	< 0.53	< 0.53	2.12	2.09	2.11	2.12
**11**	< 0.008	< 0.008	0.73	1.50	0.23	0.20
**12**	< 0.02	0.14	0.10	0.28	0.22	0.19
**13**	0.72	0.71	2.25	2.33	1.99	1.12
**14**	< 0.03	< 0.03	2.52	1.10	1.04	2.64
**15**	< 0.04	< 0.04	0.92	0.73	0.66	1.13
**16**	1.23	0.61	44.3	39.9	30.2	50.4
**17**	< 0.16	< 0.16	2.12	4.03	2.50	< 0.54
**18**	5.87	4.92	34.5	48.1	28.0	16.9
**20**	< 1.88	23.2	19.5	17.2	18.9	117
**22**	< 0.33	96.1	101	95.6	111	195
**23**	< 0.66	276	235	275	242	431
**31**	< 0.05	2.47	9.72	61.2	10.5	1.37
**32**	801	481	731	778	651	687
**33**	57.2	160	1040	1450	703	1070
**34**	< 1.19	4.48	54.5	101	64.5	18.5
**35**	< 0.61	5.30	23.1	96.2	46.9	18.2
**36**	< 0.76	< 0.76	4.45	22.96	8.18	5.63
**37**	< 0.06	< 0.06	3.14	64.54	37.13	2.16
**38**	< 0.02	< 0.02	10.6	284	267	7.09
**39**	9.50	15.2	2.27	1.33	3.53	2.20
**40**	297	374	125	63.3	114	145

**41**	37.1	26.7	105	60.9	58.5	78.2
**42**	< 0.55	15.7	166	426	201	78.2
**43**	< 0.09	46.8	134	560	180	156
**44**	< 0.02	5.44	9.66	57.6	13.9	17.9

[a] Chemical structures are given in Figure 1 and Figure 2; compounds that were not detected in any investigated sample are not shown
